# Comparative Efficacy of Continuous Ceftazidime Infusion vs. Intermittent Bolus against In Vitro Ceftazidime-Susceptible and -Resistant *Pseudomonas aeruginosa* Biofilm

**DOI:** 10.3390/antibiotics13040344

**Published:** 2024-04-09

**Authors:** Cristina El Haj, Eugènia Agustí, Eva Benavent, Laura Soldevila-Boixader, Raül Rigo-Bonnin, Fe Tubau, Benjamín Torrejón, Jaime Esteban, Oscar Murillo

**Affiliations:** 1 Infectious Diseases Service, Laboratory of Experimental Infection, Hospital Universitari de Bellvitge and Bellvitge Biomedical Research Institute, Universitat de Barcelona, 08907 Barcelona, Spain; celhaj@idibell.cat (C.E.H.); eugenia.agusti@gmail.com (E.A.); benavent.eva@gmail.com (E.B.); laura.soldeb@gmail.com (L.S.-B.); 2 Department of Clinical Laboratory, Hospital Universitari de Bellvitge and Bellvitge Biomedical Research Institute, Universitat de Barcelona, 08907 Barcelona, Spain; raulr@bellvitgehospital.cat; 3 Department of Microbiology, Hospital Universitari de Bellvitge and CIBERES-Instituto de Salud Carlos III, 08907 Barcelona, Spain; f.tubau@bellvitgehospital.cat; 4 Centres Científics i Tecnològics, Universitat de Barcelona, 08907 Barcelona, Spain; torrejonbenja@ub.edu; 5 Department of Clinical Microbiology, IIS-Fundación Jiménez Díaz, Universidad Autónoma de Madrid, 28040 Madrid, Spain; jestebanmoreno@gmail.com; 6 Centro de Investigación Biomédica en Red en Enfermedades Infecciosas (CIBERINFEC), Instituto de Salud Carlos III, 28029 Madrid, Spain

**Keywords:** beta-lactams, PK/PD, *P. aeruginosa*, biofilm, foreign-body infection

## Abstract

**Background**: As the anti-biofilm pharmacokinetic/pharmacodynamic (PK/PD) properties of antibiotics are not well-defined, we have evaluated the PK/PD indices for different regimens of ceftazidime (CAZ; with/without colistin) against *Pseudomonas aeruginosa* biofilm. **Methods**: We have used the Center for Disease Control and Prevention Biofilm Reactor with two susceptible (PAO1 and HUB-PAS) and one resistant (HUB-XDR) strains of *P. aeruginosa*. The regimens were CAZ monotherapies (mimicking a human dose of 2 g/8 h, CAZ-IB; 6 g/daily as continuous infusion at 50 mg/L, CAZ-CI_50_; and 9 g/daily at 70 mg/L, CAZ-CI_70_) and CAZ-colistin combinations. Efficacy was correlated with the CAZ PK/PD parameters. **Results**: CAZ-CI_70_ was the most effective monotherapy against CAZ-susceptible strains (Δlog CFU/mL 54–0 h = −4.15 ± 0.59 and −3.05 ± 0.5 for HUB-PAS and PAO1, respectively; *p* ≤ 0.007 vs. other monotherapies), and adding colistin improved the efficacy over CAZ monotherapy. CAZ monotherapies were ineffective against the HUB-XDR strain, and CAZ-CI_50_ plus colistin achieved higher efficacy than CAZ-IB with colistin. The PK/PD index that correlated best with anti-biofilm efficacy was *f*AUC_0–24h_/MIC (r^2^ = 0.78). **Conclusions**: CAZ exhibited dose-dependent anti-biofilm killing against *P. aeruginosa*, which was better explained by the *f*AUC_0–24h_/MIC index. CAZ-CI provided benefits compared to CAZ-IB, particularly when using higher doses and together with colistin. CAZ monotherapies were ineffective against the CAZ-resistant strain, independently of the optimized strategy and only CAZ-CI plus colistin appeared useful for clinical practice.

## 1. Introduction

Antibiotic therapy for biofilm-related infections represents a challenge for clinicians [1,2]; thus, optimizing the use of antimicrobials in this setting is recommended. However, the conventional pharmacokinetic/pharmacodynamic (PK/PD) indices of antibiotic efficacy were defined by using planktonic bacteria and have not proved useful for guiding the treatment of biofilm-related infections [3].

The proportion of osteoarticular and orthopedic device-related infections caused by Gram-negative bacilli (GNB) has increased progressively over recent years, with infections caused by *Pseudomonas aeruginosa* being of great concern due to their ability to form biofilms and acquire resistance mechanisms [4]. Beta-lactams still remain a viable first-line therapy for osteoarticular infections, including those caused by multidrug-resistant GNB [5]. Locally, ceftazidime (CAZ) is still a valuable option against most *P. aeruginosa* strains; however, almost 15–17% of these isolates are multi-drug or extremely-drug resistant, mainly belonging to high-risk clones [6]. Beta-lactams have traditionally been considered to have poor activity against biofilms as they are principally active against bacteria in the growth phase (planktonic) [7]. Particularly, the time-dependent killing of beta-lactams has been questioned against bacterial biofilms based on results from pre-clinical works that mainly evaluated the efficacy of ceftazidime and meropenem [8,9,10]. Overall, few previous works have focused on the evaluation of specific PK/PD indices of beta-lactams used in biofilm-related infections or have suggested the most appropriate dosage and type of administration in clinical settings to improve their efficacy in the treatment of device-related infections [11,12]. 

In vitro dynamic models, such as the Center for Disease Control and Prevention Biofilm Reactor (CBR), allow the PK/PD parameters of antimicrobials against bacterial biofilms to be mimicked over time. It has already been validated and used to evaluate the efficacy of different antimicrobials against biofilm-embedded bacteria, including several *P. aeruginosa* strains [10,13]. In our previous works, we evaluated the PK/PD indices of meropenem, predicting anti-biofilm efficacy against several meropenem-susceptible and -resistant *P. aeruginosa* strains [14]. Also, we performed a partial evaluation of the efficacy of CAZ administered in continuous infusion at a range of drug concentrations equivalent to clinical doses lower than 6 g/daily (4 mg/L to 40 mg/L) against CAZ-susceptible *P. aeruginosa* strains [10]. With this in mind, in the present work we used the same CBR model to further explore the anti-biofilm PK/PD indices of CAZ against *P. aeruginosa* isolates. For this aim, we evaluated the efficacy of (i) two regimens of CAZ administration (intermittent bolus and continuous infusion), (ii) doses equivalent to the highest dosages used in clinical practice (6 g daily and 9 g daily), and (iii) the efficacy against two CAZ-susceptible *P. aeruginosa* strains (HUB-PAS and PAO1) and one CAZ-resistant *P. aeruginosa* strain (HUB-XDR). We also studied the anti-biofilm effect of CAZ plus colistin.

## 2. Results

All the minimum inhibitory concentrations (MICs), minimum biofilm inhibitory concentrations (MBICs), and minimum biofilm eradication concentrations (MBECs) for CAZ and colistin are shown in Table 1. The MIC values of CAZ were 1, 2, and 32 mg/L for HUB-PAS, PAO1, and HUB-XDR, respectively.

### 2.1. PK/PD Analyses of CAZ Monotherapies

At the start of the therapeutic experiments (0 h), the biofilm-embedded bacterial counts for each strain (log(10) CFU/mL ± SD) were 6.53 ± 0.45, 6.76 ± 0.34, and 6.8 ± 0.41 for HUB-PAS, PAO1, and HUB-XDR, respectively. 

The regimens used were CAZ equivalent to human doses of 6 g daily administered by intermittent bolus (IB), 6 g daily (50 mg/L) in continuous infusion (CAZ-CI_50_), or 9 g daily (70 mg/L) in continuous infusion (CAZ-CI_70_), against CAZ-susceptible strains (HUB-PAS and PAO1) and a CAZ-resistant *P. aeruginosa* strain (HUB-XDR).

**Figure 1:**
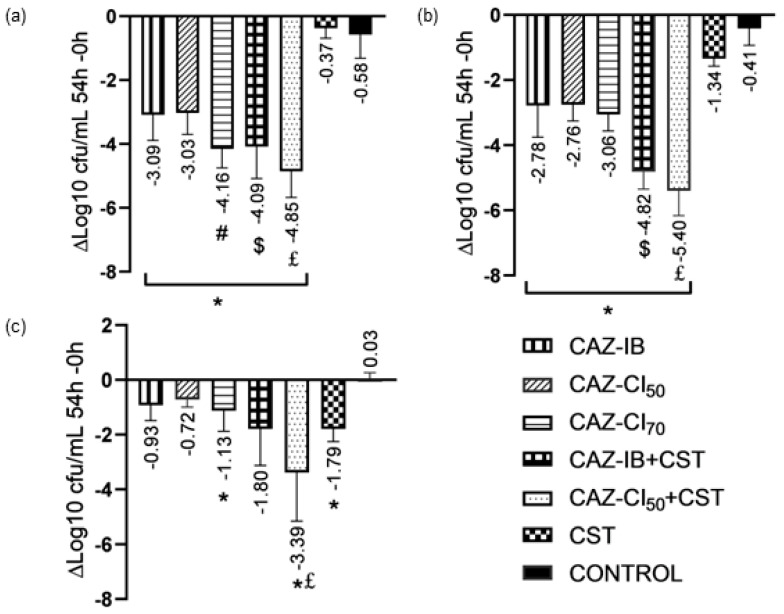
Antibiotic efficacies against *P. aeruginosa* biofilm at 54 h. Decrease in bacterial counts between 54 h and 0 h in CBR for HUB-PAS (**a**), PAO1 (**b**), and HUB-XDR (**c**) strains using different CAZ regimens, CST, and the combination of CAZ + CST. The results are expressed using the log change method (mean ± SD). n = 12. Statistical analyses are shown for each strain as follows: * *p* ≤ 0.02 vs. control groups; # *p* ≤ 0.007 vs. CAZ-IB and CAZ-CI_50_; $ *p* ≤ 0.038 vs. CAZ-IB; £ *p* ≤ 0.001 vs. CAZ-CI_50._ Abbreviations: CAZ-IB (parallelly striped bars), intermittent bolus of ceftazidime; CAZ-IC_50_ (diagonally striped bars), continuous infusion of CAZ at 50 mg/L; CA-CI_70_ (horizontally striped bars), continuous infusion of CAZ at 70 mg/L; CAZ-IB + CST (open squares bars) intermittent bolus of CAZ plus colistin; CAZ-IC_50_ + CST (dotted bars) continuous infusion of CAZ at 50 mg/L plus colistin; CST (filled and opened squares bars), colistin; control (filled bars); CFU, colony-forming unit.

Against CAZ-susceptible strains (HUB-PAS and PAO1), all CAZ monotherapies (CAZ-IB, CAZ-CI_50_, and CAZ-CI_70_) presented greater efficacy than the control, with CAZ-CI_70_ being the most active treatment against each strain, achieving a bactericidal activity (Figure 1a,b). 

Against the CAZ-resistant strain (HUB-XDR), all CAZ monotherapies had reduced efficacy with only CAZ-CI_70_ showing a significantly higher decrease in bacterial counts than the control (Figure 1c).

Overall, only the CAZ-CI_70_ regimen could completely avoid the appearance of CAZ-resistant strains. 

**Figure 2:**
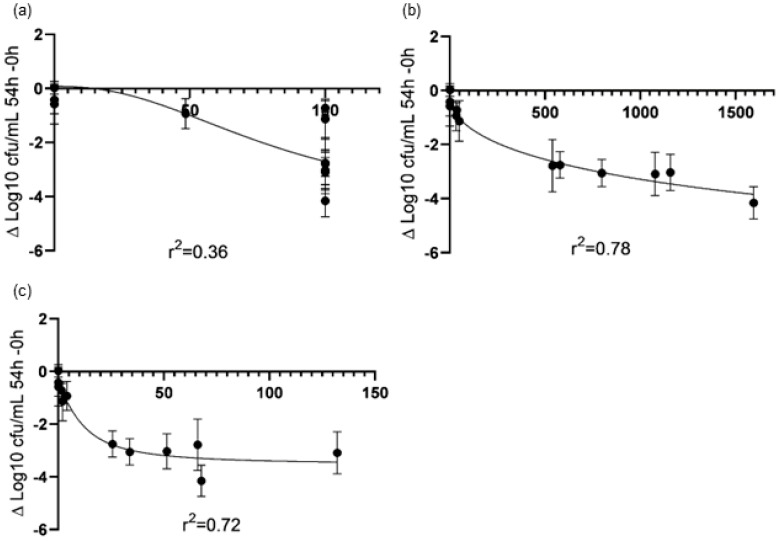
Correlation between PK/PD indices of therapeutic regimens of CAZ (*f*%T > MIC (**a**), *f*AUC/MIC (**b**), and *f*C_max_/MIC (**c**)) and anti-biofilm efficacy against each strain. Abbreviations: *f*AUC, area under the concentration–time curve; CFU, colony-forming unit; MIC, minimum inhibitory concentration; *f*C_max_, peak concentration; *f*%T > MIC, time the free antimicrobial concentration remains above the MIC.

Table 2 shows the main PK/PD indices obtained for the different CAZ regimens (IB, IC_50_*,* and IC_70_) against each strain. All treatments maintained CAZ concentrations above the MIC throughout treatment (*f*%T > MIC = 100%) except for the CAZ-IB regimen for HUB-XDR (*f*%T > MIC = 48.5%), which has the highest MIC. The *f*AUC_0–24_, and therefore *f*AUC_0–24h_/MIC values, were higher for the CAZ-CI_70_ regimen than for CAZ-IB and CAZ-CI_50_, but the greatest *f*C_max_ was achieved by CAZ-IB. The PK/PD index that best correlated with the anti-biofilm efficacy of CAZ was the *f*AUC_0–24h_/MIC (r^2^ = 0.78). Figure 2 shows the correlation between the main PK/PD indices and the efficacy for each regimen.

### 2.2. Combination Therapy with Colistin

Colistin monotherapy had limited efficacy against CAZ-susceptible strains at 54 h (Δlog CFU/mL 54–0 h = −0.37 ± 0.31 and −1.34 ± 0.23 for HUB-PAS and PAO1, respectively; Figure 1a,b). However, it presented the highest activity among monotherapies against HUB-XDR (−1.79 ± 0.46 vs. control; *p* = 0.01; Figure 1c). Regrowth was observed in all cases.

The addition of colistin improved the efficacy of all CAZ monotherapies, and achieved with this combined therapy a bactericidal effect against all strains (except for CAZ-IB plus colistin against HUB-XDR). Overall, the combination CAZ-CI_50_ plus colistin demonstrated bactericidal activity higher than that of CAZ-IB plus colistin against CAZ-susceptible and CAZ-resistant *P. aeruginosa* strains. In all cases, combination therapy also prevented the emergence of colistin-resistant and CAZ-resistant strains.

### 2.3. Confocal Laser Scanning Microscopy

An excerpt of representative images is shown in Figure S1 (Supplementary Data). At 0 h, the biofilm thickness of the HUB-PAS strain was 14.186 ± 0.03 µm, and the percentage of live cells was 97.13%. After treatment using CAZ-IB, CAZ-CI_50_, and CAZ-CI_70_, the biofilm was 20.864 ± 0.43, 13.8225 ± 0.03, and 8.356 ± 2.13 µm thick, respectively. The percentage of live cells was higher in biofilms exposed to CAZ-IB (32.17%) and CAZ-CI_50_ (26.08%) than in those treated with CAZ-CI_70_ (14.72%).

## 3. Discussion

There is still a need to evaluate the PK/PD indices most useful for predicting the efficacy of beta-lactams against biofilm-related infections, because traditional parameters were defined to achieve an *f*%T > MIC above 40–60% and a drug concentration approximately 3–4 times the MIC value for planktonic bacteria and not taking the particularities of biofilm-embedded bacteria into account [3,15]. In the present work, we evaluated the PK/PD parameters of several CAZ regimens equivalent to the maximum dosages used in humans (6 g and 9 g daily) administered by IB and CI, against CAZ-susceptible and CAZ-resistant *P. aeruginosa* strains with different MIC values. The *f*%T > MIC index, considered the most important PK/PD index for beta-lactams, was optimized to its maximum (100%) in all cases but the CAZ-IB regimen for the HUB-XDR strain (48.5%; Table 2). However, under these conditions, we observed notable differences between the efficacy of CAZ, with and without colistin, which depended on the strain susceptibility, the dosage, and the form of administration. Overall, CAZ monotherapies achieved poor efficacy against the HUB-XDR strain (Figure 1c), whereas relevant and sometimes bactericidal activity was observed for CAZ-susceptible strains, although benefits were noted when using CAZ-CI at usual (CAZ-CI_50_) and at high doses (CAZ-CI_70_) compared to CAZ-IB (Figure 1a,b). Specifically, CAZ-CI_70_ monotherapy showed the greatest efficacy, being significantly higher than CAZ-CI_50_ or CAZ-IB against the HUB-PAS strain, which had the lowest MIC value (Figure 1a). When comparing the activity of CAZ-IB and CAZ-CI_50_, which were equivalent to a human dose of 6 g daily, the benefits of CI were mainly observed in combination with colistin for all strains.

To our knowledge, few studies have evaluated the specific PK parameters of CAZ against biofilms. Hengzhuang et al. published three works using in vitro methods with planktonic and biofilm bacteria and an in vivo model of biofilm lung infection formed by the PAO1 strain and some derived mutants. In these works, the authors noted a change in beta-lactam (imipenem and CAZ) PD values in biofilms compared to the time-dependent killing in planktonic bacteria, and therefore, the role of dose in these infections [8,16]. They observed that CAZ exhibited concentration-dependent killing that was more evident against a beta-lactamase-overproducing mutant strain [9]. Our group used the CBR model to evaluate the efficacy of CAZ using CI at concentrations of 4 to 40 mg/L against two CAZ-susceptible *P. aeruginosa* strains, mimicking concentrations allowed in clinical practice with doses lower than 6 g/d, revealing an improved anti-biofilm activity when higher concentrations of CAZ were used for longer periods [10]. In the present work, we performed additional and complementary experiments to evaluate in depth the anti-biofilm PK/PD indices of CAZ; concisely, compared to our previous works, herein we evaluated (i) the comparative efficacy of ceftazidime administered by intermittent bolus or in continuous infusion, (ii) the efficacy of ceftazidime administered at the highest dosages used in clinical practice (6 g daily and 9 g daily), and (iii) the ceftazidime efficacy against ceftazidime-susceptible and -resistant *P. aeruginosa* strains. Overall, the present work highlights how to optimize the PD parameters of CAZ therapy. 

The efficacy of CAZ against *P. aeruginosa*, when administered by IB and CI, has been compared in a few experimental works, but none has evaluated activity on biofilm cells. By using a two-compartment in vitro PD model, Cappelletty et al. compared the activity of CAZ-IB (equivalent to 2 g/8 h) and CAZ-CI at 5, 10, and 20 mg/L against one CAZ-susceptible and one CAZ-resistant strain. This showed that CAZ at 20 mg/L had a significantly greater efficacy than the other CI regimens and with similar activity to CAZ-IB against the CAZ-susceptible strain, despite the CI regimen (AUC_0–24h_ values = 677) mimicking a lower dose than the IB regimen (AUC_0–24h_ = 1780). Moreover, all monotherapies were ineffective against the resistant strain [17]. In another work, Mouton et al. used a similar in vitro model to compare the activity of the same dose of CAZ (300 mg/L/24 h), administered by CI or IB against three *P. aeruginosa* strains. They noted that CI exposed the strains to high and sustained CAZ concentrations and thus confirmed that this regimen was the most effective [18].

Our results mostly agree with these previous studies by Cappelletty et al. and Mouton et al., though we emphasize that the *f*AUC/MIC ratio, and not the *f*%T > MIC, was the PK/PD index that best correlated with CAZ efficacy in biofilm cells. We found that the *f*AUC/MIC ratio could be optimized by using high doses and CI administration. In fact, this index has been related to the efficacy of most antibiotics, considered either time-dependent killing or concentration-dependent killing agents, and an AUIC value of 125 (which represents near 80% of the AUC above the MIC) is a key point associated with efficacy and resistance being overcome [19,20,21]. Our findings of ineffective CAZ monotherapies against the HUB-XDR strain are of particular interest here. Despite CAZ-IB, CAZ-CI_50_, and CAZ-CI_70_ having *f*%T > MIC values of 48.5%, 100%, and 100%, respectively (Figure 2a), the *f*AUC/MIC values were clearly below the cut-off in all cases (Figure 2b). While awaiting further studies, these results support the position that CAZ monotherapy, independent of the optimal dosage and form of administration, is not a valid therapeutic alternative to treat biofilms formed by CAZ-resistant *P. aeruginosa* strains. Given the recent appearance of other beta-lactams that can be used alone or in combination with beta-lactamase inhibitors, their efficacy against biofilm-related infections should be evaluated [22,23,24].

Colistin has been reported to have a good activity against biofilm cells, and in particular, the combination of beta-lactams plus colistin appears to be highly effective against biofilms formed by *P. aeruginosa* [8,25]. Consistent with previous works, our results again show the synergistic effects of CAZ with colistin, with this combination resulting in the best therapy. Overall, the intravenous administration of beta-lactams to allow high drug serum concentrations, as well as their use in combination with colistin, represent two therapeutic strategies with proven therapeutic efficacy in clinical settings with osteoarticular and orthopedic device-related infections caused by GNB.

There are some limitations in the present study, mainly concerning the use of the in vitro biofilm model. Although the CBR allows the evaluation of antibiotic PD parameters against biofilms, this complex structure may differ from in vivo biofilm infections. Furthermore, the model did not mimic other environmental variables, such as host–pathogen interactions. The use of only three strains may preclude study generalization to all *P. aeruginosa*; however, the present work is still of interest due to its evaluation of CAZ PD parameters against susceptible and resistant strains. The synergistic effects provided by the combination of colistin plus ceftazidime at the highest concentration (70 mg/L) could also be beneficial and should be further explored. Finally, we note that in vitro models can induce the emergence of resistant strains, which may also affect the efficacy of antibiotics, particularly over a prolonged therapy.

In conclusion, CAZ exhibited dose-dependent anti-biofilm killing of *P. aeruginosa* strains, which was best explained by the *f*AUC/MIC index. The administration of CAZ by CI provided benefits compared to CAZ by IB, particularly when using high doses alone and in combination with colistin. However, CAZ monotherapy was ineffective against biofilms formed by CAZ-resistant *P. aeruginosa* strains, independent of dosage optimization; in this case, only colistin plus CAZ administered by CI appeared useful. These findings not only show the potential to optimize CAZ therapy against biofilm-related infections due to *P. aeruginosa* but also demonstrate its limited activity against CAZ-resistant strains.

## 4. Materials and Methods

### 4.1. Bacterial Isolates and Antimicrobial Agents

Three *P. aeruginosa* strains were used. The first was the reference PAO1 strain (American Type Culture Collection, Rockville, MD, USA). The second was the clinical isolate HUB-PAS (from a case of prosthetic joint infection at Hospital Universitari Bellvitge). Both strains were susceptible to CAZ, but with different minimum inhibitory concentrations (MICs). The third strain, HUB-XDR, belonged to the high-risk clone ST-175 that has shown extensive drug resistance (AmpC ß–lactamase hyperproduction plus OprD porin deletion), including resistance to CAZ. All strains were susceptible to colistin.

Ceftazidime and colistin were purchased from manufacturers and the purified powder for each antibiotic was resuspended following laboratory recommendations.

### 4.2. Determination of Minimum Inhibitory Concentrations, Minimum Biofilm Inhibitory Concentrations, and Eradication Concentrations

The MICs of CAZ and colistin were determined by broth microdilution, following standard recommendations [26]. The MBICs and the MBECs were determined using an MBEC device (Innovotech Inc., Edmonton, AB, Canada) [27] with a range concentration from 0.5 mg/L to 512 mg/L of each antibiotic. All experiments were performed at least in triplicate.

### 4.3. In Vitro Pharmacokinetic/Pharmacodynamic Biofilm Model

A CDC Biofilm Reactor system (CBR; BioSurface Technologies Corp., Bozeman, MT, USA) was used based on a previously described protocol [10,13], which consisted of a biofilm conditioning phase for 48 h followed by a therapeutic phase for 54 h. After the biofilm conditioning phase, where biofilm was formed in Teflon coupons, the therapeutic phase started (baseline, 0 h) with fresh medium (free or with CAZ) was pumped at a flow rate of 2 mL/min to reproduce the half-life of CAZ (t ½ = 2 h).

The following therapeutic regimens were evaluated, mimicking human dosages: CAZ by intermittent bolus (CAZ-IB; free C_max_ = 134 mg/L), equivalent to 2 g every 8 h in humans; CAZ by continuous infusion of 50 mg/L (CAZ-CI_50_), equivalent to 6 g/24 h; and CAZ by continuous infusion of 70 mg/L (CAZ-CI_70_), equivalent to 9 g/24 h [28,29]. We also evaluated colistin at 3.50 mg/L by CI, simulating the unbound plasma steady-state concentration observed in patients receiving 6–9 MU/day of colistin methanesulfonate [30]. Flow rates were calibrated before each experiment and monitored throughout to ensure optimal system operation. We evaluated control therapy (no antibiotic), monotherapy (CAZ-IB, CAZ-CI_50_, CAZ-CI_70_ and colistin), and combination therapy (colistin plus CAZ-CI_50_ and colistin plus CAZ-IB). All experiments were performed at least in duplicate.

### 4.4. Pharmacokinetic/Pharmacodynamic Analysis

For the PD analyses, at least three coupon samples (biofilm-embedded bacteria) were collected at 0, 6, 24, 30, 48, and 54 h. The removed coupons were then processed as described elsewhere [10], with the coupon samples serially diluted (10-fold), plated on agar plates (Beckton Dickinson, Barcelona, Spain), and incubated at 37 °C for 24–48 h. Bacterial counts were expressed as log 10 CFU/mL and the efficacy was evaluated as a decrease in bacterial counts (Δlog 10 CFU/mL X–0 h). The activity of monotherapy or combination regimens was defined as bactericidal or bacteriostatic, synergistic or antagonistic based on standard definitions [31].

The emergence of resistance to CAZ and colistin was screened during the treatment. Samples recovered from coupons were plated into agar plates containing 8 mg/L of CAZ and 4 mg/L of colistin and incubated over 24–48 h. After incubation, MICs were determined following standard protocol [26]. 

To measure the concentrations of CAZ and colistin, 1 mL of medium containing antibiotic was collected from the CBR system at different times, placed in 1.5 mL microcentrifuge tubes, and stored immediately at −80 °C. We used high-performance liquid chromatography to determine antibiotic levels, as previously described [10].

### 4.5. Confocal Laser Scanning Microscopy

Coupons were evaluated by confocal laser scanning microscopy to confirm biofilm infection (0 h) and treatment activity (54 h), according to a previously reported protocol [10].

### 4.6. Statistical Analysis

Data were analyzed using IBM SPSS v.20.0 (IBM Corp., Armonk, NY, USA). The efficacy of each therapy was evaluated using the log change method from 0 h to each time-point (Δlog CFU/mL X–0 h). Therapeutic groups were compared by analysis of variance and Tukey’s post-hoc test per treatment. Differences were considered statistically significant at a *p*-value of <0.05.

The PK parameters, PK/PD indices, and their correlation with the efficacy of each therapeutic regimen were determined according to previously reported methodology [14]. Briefly, the included PK parameters, considering free-drug concentrations, were as follows: elimination half-life (t ½), peak concentration (*f*C_max_) for IB, steady-state concentration (*f*Css) for CI, and area under the concentration–time curve (*f*AUC_0-t_). For PK/PD integration of CAZ, we calculated the area under the curve for 24 h versus MIC (*f*AUC_0–24h_/MIC), the free-drug peak concentration versus MIC (*f*C_max_/MIC), and the percentage of time during which the free-drug level exceeded the MIC (*f*%T > MIC). The efficacy of the therapeutic regimens at the end of experiments (Δlog CFU/mL 54–0 h) was correlated with these PK/PD indices for each CAZ regimen and used to generate fit curves. 

## Figures and Tables

**Table 1:** Minimum inhibitory concentrations (MICs), minimum biofilm inhibitory concentration (MBICs), and minimum biofilm eradication concentration (MBECs) for the *Pseudomonas aeruginosa* strains examined in this study.

Strain	MIC (mg/L)		MBIC (mg/L)		MBEC (mg/L)	
	CAZ	CST	CAZ	CST	CAZ	CST
HUB-PAS	1	1	4	16–8	>512	>512
PAO1	2	0.5	8	32	>512	>512
HUB-XDR	32	1	64	16	>512	>512

Higher concentrations than 512 mg/L were not evaluated for CAZ and CST. Abbreviations: CAZ, ceftazidime; CST, colistin; MBIC; minimum biofilm inhibitory concentration; MBEC, minimum biofilm eradication concentration; and MIC, minimum inhibitory concentration.

**Table 2:** Pharmacokinetic parameters of each CAZ regimen for each strain used.

		*f*%T > MIC (%)			*f*AUC_0–24h_/MIC			*f*C_max_/MIC		
	Strain	HUB-PAS	PAO1	HUB-XDR	HUB-PAS	PAO1	HUB-XDR	HUB-PAS	PAO1	HUB-XDR
CAZ regimen										
IB		100	100	48.5	1078.34 ± 136.75	539.17 ± 68.38	33.69 ± 4.27	132.16 ± 5.49	66.08 ± 2.74	4.13 ± 0.17
CI_50_		100	100	100	1157.10 ± 61.75	578.55 ± 30.87	36.15 ± 1.92	51.45 ± 6.3	25.72 ± 3.15	1.6 ± 0.19
CI_70_		100	100	100	1594.59 ± 96.43	797.29 ± 48.22	49.83 ± 3.01	67.75 ± 4.15	33.87 ± 2.07	2.11 ± 0.19

Abbreviations: *f*AUC_0–24h_, area under the curve for 24 h; CI, continuous infusion, *f*C_max_, free-drug peak concentration; IB, intermittent bolus; minimum inhibitory concentration, MIC and *f*%T > MIC, percentage of time during which the free-drug level exceeded the MIC.

## Data Availability

Data are contained within the article and Supplementary Materials.

## Supplementary Materials

The following supporting information can be downloaded at: https://www.mdpi.com/article/10.3390/antibiotics13040344/s1, Figure S1: Representative confocal images of HUB-PAS biofilm before and after treatment. Control (0 h), (a); HUB-PAS CAZ-IB (54 h), (b); HUB-PAS CAZ-CI_50_ (54 h), (c); HUB-PAS CAZ-CI_70_ (54 h), (d).

## Acronyms

CAZ	ceftazidime
CBR	Center for Disease Control and Prevention Biofilm Reactor
CDC	Center for Disease Control and Prevention
CFU	colony-forming unit
CI	continuous infusion
CST	colistin
*f*AUC_0–24h_	free area under the curve from 0 to 24 h
*f*C_max_	peak concentration
*f*Css	steady-state concentration
*f*%T	the percentage of time
GNB	Gram-negative bacilli
IB	intermittent bolus
MBEC	minimum biofilm eradication concentration
MBIC	minimum biofilm inhibitory concentration
MIC	minimum inhibitory concentration
PD	pharmacodynamic
PK	pharmacokinetic
t ½	elimination half-life
