# Supplementary material for: Comparative Efficacy of Continuous Ceftazidime Infusion vs. Intermittent Bolus against In Vitro Ceftazidime-Susceptible and -Resistant Pseudomonas aeruginosa Biofilm
Source: Antibiotics (Basel). 2024 Apr 9;13(4):344. doi: 10.3390/antibiotics13040344 (PMC11047404; doi:10.3390/antibiotics13040344)
Supplement: Supplementary file 1 [file antibiotics-13-00344-s001.zip › antibiotics-2940776-supplementary.pdf]

Supplementary data

Figure S1. Representative confocal images of HUB-PAS biofilm before and after treatment. Control (0h), a; HUB-PAS CAZ-IB (54h), b; HUB-PAS CAZ-CI$_{50}$ (54h), c; HUB-PAS CAZ-CI$_{70}$ (54h), d.

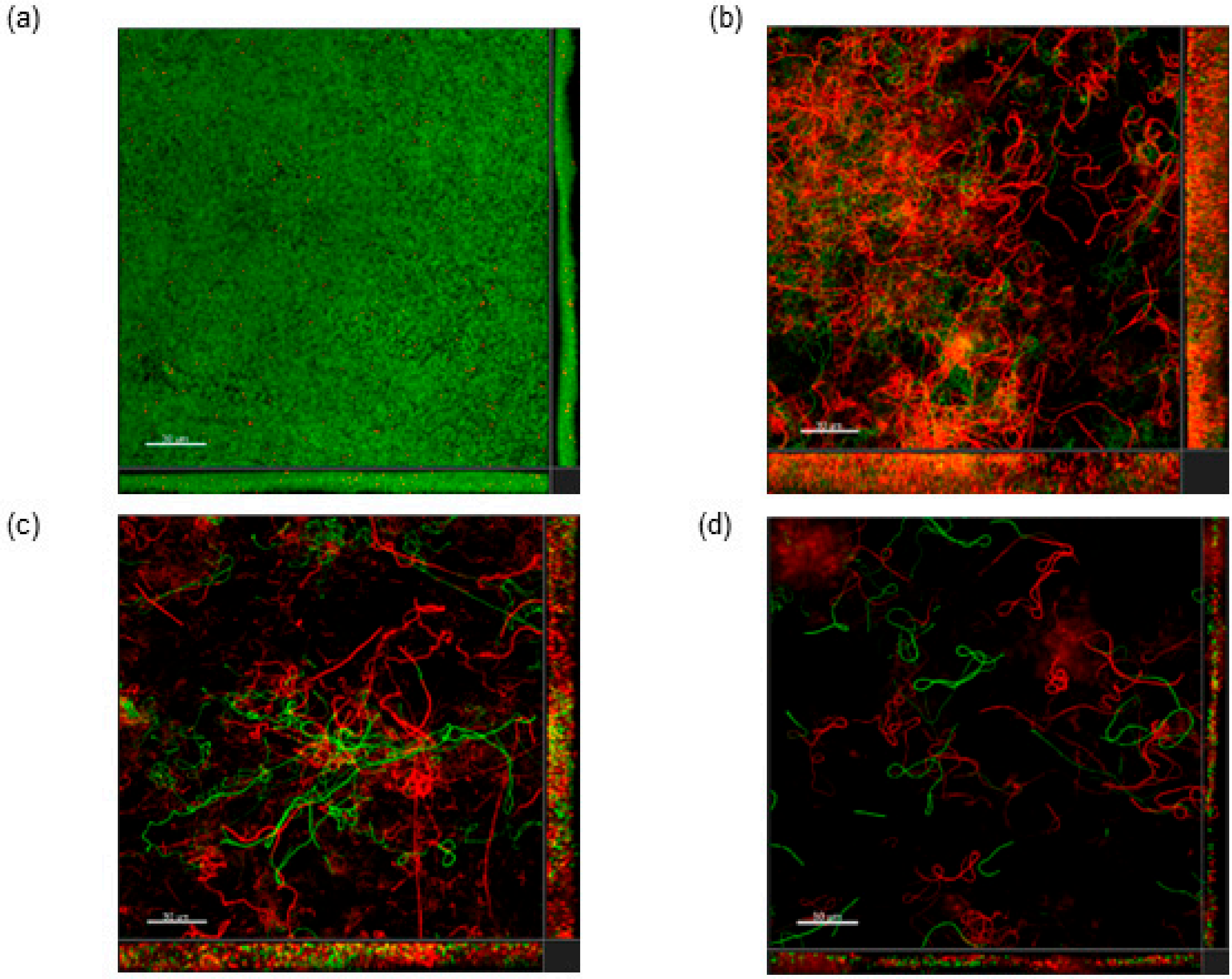

Abbreviations: CAZ, ceftadizime; CI, continuous infusion and IB, intermittent bolus
